# Erratum: search-related suppression of hippocampus and default network activity during associative memory retrieval

**DOI:** 10.3389/fnhum.2013.00002

**Published:** 2013-01-24

**Authors:** Emilie T. Reas, Sarah I. Gimbel, Jena B. Hales, James B. Brewer

**Affiliations:** ^1^Department of Neurosciences, University of CaliforniaSan Diego, La Jolla, CA, USA; ^2^Department of Radiology, University of CaliforniaSan Diego, La Jolla, CA, USA

**A commentary on**

**Search-related suppression of hippocampus and default network activity during associative memory retrieval**

by Reas, E. T., Gimbel, S. I., Hales, J. B., and Brewer, J. B. (2011). Front. Hum. Neurosci. 5:112. doi: 10.3389/fnhum.2011.00112

Recent work using an analysis technique employed in this study has led us to realize that a section of our results should be restated. The following revised paragraph should replace the originally published paragraph on page 7 of our article, under the heading “Activity Correlations with Response Time.”

## Activity correlations with response time

To examine a possible relationship between response fluency and hippocampal and default network suppression, correlations between BOLD signal and response time were computed. Activity was negatively correlated with response time (*p* < 0.05) during both the classify and the recall tasks in bilateral superior temporal cortex and PCC. During only the recall, but not the classify task, negative correlations with response time were additionally observed in bilateral anterior hippocampus, medial PFC, and inferior parietal cortex. Thus, greater activity in these regions was correlated with a faster response time.

